# Shaping a research-driven future for speech-language therapy and audiology in South Africa

**DOI:** 10.4102/sajcd.v72i1.1159

**Published:** 2025-10-31

**Authors:** Anita Edwards, Anna-Mari Olivier, Faheema Mahomed-Asmail, Jeannie van der Linde

**Affiliations:** 1 South African Speech Language and Hearing Association, Durban, South Africa; 2 Private Practice, Speech Language Therapist, George, South Africa; 3 Department of Speech-Language Pathology and Audiology, Faculty of Humanities, University of Pretoria, Pretoria, South Africa

## Introduction

The *South African Journal of Communication Disorders* (SAJCD) publishes research articles and papers that critically evaluate theoretical, philosophical, and conceptual issues dealing with human communication and its disorders, including dysphagia, service provision, training, and policy. In line with this scope, Vol 72 No 1 has seen an increase in the submission of opinion pieces, clinical perspective contributions, and review articles, which may indicate a fresh interest in publication processes and the dissemination of research practices. The increased number of review articles, in particular, has prompted the editorial team to revisit the article categories and reviewer forms, ensuring closer alignment with evolving methodological developments in the this type of manuscript submission and reaffirming the journal’s commitment to continual improvement in supporting authors. The focus throughout is on cultivating locally relevant research, equitable service models, and interdisciplinary integration, tailored to the sociocultural context.

## Key themes and implications

### Hearing loss

The three articles and one opinion paper relating to hearing loss collectively expand the discourse on paediatric audiology, calling for the integration of audiological monitoring in the public health context and providing evidence on the feasibility of improved models for newborn hearing screening by including task-shifting to nurses. Linked to hearing screening the validated of speech-in-noise test for Swahili-speaking populations was investigated, supporting early identification in under-resourced regions. The unique needs of hard-of-hearing learners within deaf education were explored in the third paper, providing empirical data on dual educational challenges, which can guide differentiated support in specialised schools. The included opinion piece advocates for the integration of routine audiological assessments within maternal and paediatric human immunodeficiency virus (HIV) healthcare services, highlighting the need for structural changes in policy to support preventive audiological care.

### Multilingual language

On the topic of multilingual language, the two articles offer important interdisciplinary concepts. The concept of translanguaging as a culturally responsive therapeutic tool for aphasia rehabilitation promotes inclusive communication strategies in multilingual communities, fostering dignity and participation. The description of the language use by younger generations in Pedi families expands pragmatic research in African languages, contributing to sociolinguistics and culturally relevant speech-language therapy practice.

### Swallowing and feeding and dysphagia

The three articles on this topic address the opposite ends of the life cycle. Two of the articles address maternal and child health by discussing the role of speech-language therapists (SLTs) in supporting breastfeeding, with benefits for infant nutrition and bonding as well as reporting on early intervention and monitoring strategies for high-risk infants that contribute to improved survival-to-thriving pathways. The third related article raises awareness of dysphagia in the ageing populations, advocating for early screening in this population.

These articles illustrate the role of SLTs in interdisciplinary perinatal care and add longitudinal data on developmental outcomes. In addition, the research informs person-centred geriatric care by linking patient self-perceptions with clinical data.

### Training and workplace

The two articles that relate to the journal focus on training and examining students’ perspectives on corporate practice. The articles offer critical reflection on curriculum reform, aligning training with South Africa’s socio-cultural realities and exploring our professional roles in non-traditional settings, for example, the corporate environment, thereby broadening SLTs’ impact into workplace wellness by enhancing workplace communication and interpersonal skills.

### Looking ahead

Digital transformation of our practice models has become an important area of research interest and tele-practice will continue to be at the forefront of development if we are to provide the needed services to our clients. The article which validates telehealth as viable for fluency disorders adds to the field by providing evidence for user-centred telepractice and by informing ethical and effective remote stuttering intervention.

The opinion paper ‘Setting a Research Agenda for Speech Therapy and Audiology Practice’ underscores the necessity of a structured research agenda to drive meaningful changes in clinical practice in South Africa. In order to address the unique needs of South African patients, this opinion piece invites practitioners, policymakers and researchers to collaborate, define and prioritise research topics.

## Conclusion

Thank you to the authors, reviewers, and editors, whose work anchors the vision of this edition. Volume 72 No 1 exemplifies locally grounded scholarship and charts a transformative research and practice pathway for speech-language therapy and audiology rooted in South Africa’s realities and committed to equitable, evidence-informed progress.

